# A cross-sectional study of gastrointestinal symptoms, depressive symptoms and trait anxiety in young adults

**DOI:** 10.1186/s12888-020-02940-2

**Published:** 2020-11-11

**Authors:** Fanny Söderquist, Mikaela Syk, David Just, Zorana Kurbalija Novicic, Annica J. Rasmusson, Per M. Hellström, Mia Ramklint, Janet L. Cunningham

**Affiliations:** 1 Department of Neuroscience, Psychiatry Uppsala University, Uppsala, Sweden; 2 Department of Medical Sciences, Gastroenterology/Hepatology, Uppsala University, Uppsala, Sweden

**Keywords:** Irritable bowel syndromes, Depressive disorder, Anxiety disorder, Personality

## Abstract

**Background:**

>Patients with functional gastrointestinal disorders have a high psychiatric co-morbidity. This study aimed to investigate and characterise gastrointestinal symptoms in relation to depressive symptoms and trait anxiety in a well-defined population of young adult psychiatric outpatients and healthy controls.

**Methods:**

Gastrointestinal symptoms were assessed with the Gastrointestinal Symptom Rating Scale for Irritable Bowel Syndrome (GSRS-IBS). Depressive symptoms were assessed with the Montgomery-Åsberg Depression Rating Scale- Self assessment (MADRS-S). Trait anxiety was estimated with three of the Swedish universities of Personality (SSP) scales: Somatic trait anxiety, Psychic trait anxiety and Stress susceptibility. Self-ratings were collected from 491 young adult psychiatric outpatients and 85 healthy controls. Gastrointestinal symptom severity was compared between patients with and without current psychotropic medication and controls. Associations between gastrointestinal symptoms, depressive symptoms and trait anxiety were assessed using Spearman’s coefficients and generalized linear models adjusting for possible confounders (sex, body mass index, bulimia nervosa).

**Results:**

Patients, with and without current psychotropic medication, reported significantly more gastrointestinal symptoms than controls. In the generalized linear models, total MADRS-S score (*p* < 0.001), Somatic trait anxiety (*p* < 0.001), Psychic trait anxiety (*p* = 0.002) and Stress susceptibility (*p* = 0.002) were independent predictors of the total GSRS-IBS score. Further exploratory analysis using unsupervised learning revealed a diverse spectrum of symptoms that clustered into six groups.

**Conclusion:**

Gastrointestinal symptoms are both highly prevalent and diverse in young adult psychiatric outpatients, regardless of current psychotropic medication. Depressive symptom severity and degree of trait anxiety are independently related to the total gastrointestinal symptom burden.

**Supplementary Information:**

The online version contains supplementary material available at 10.1186/s12888-020-02940-2.

## Background

The GI tract and the brain are intimately connected via bidirectional neural, endocrine and immune pathways, commonly referred to as the gut-brain axis (GBA) [1]. This complex communication system not only ensures GI homeostasis but also influences motivation and higher cognitive functions. The GBA includes the central nervous system, the autonomic nervous system, the enteric nervous system and the hypothalamic-pituitary-adrenal (HPA) axis, as well as signalling via gut peptides [2]. The role of the GBA is to integrate gut functions and link emotional and cognitive centres of the brain with peripheral intestinal functions and mechanisms such as appetite, satiety, immune activation, intestinal permeability, enteric reflexes and enteroendocrine signalling [1].

Functional gastrointestinal disorders (FGIDs) are a group of disorders affecting the GI tract, often causing considerable impact on health-related quality of life, but where there is no clear pathogenesis. The most common FGID is irritable bowel syndrome (IBS), which affects the lower GI tract [3]. Psychiatric co-morbidity is high in patients with FGIDs, suggesting shared or interacting disease mechanisms, possibly related to the proposed communication between the gut and brain, in which variations of the intestinal microbiome may play an essential role [4, 5]. Specifically, a recent review points out an alternative pathway from the gut (with its microbiome) via the vagus nerve and the mechanisms of the bidirectional communication between the gut and brain for the development of depression [6]. In another report, a high correlation between stress-related mental symptoms, including anxiety and IBS was brought forward, providing a basis for further studies of the vagus nerve and the GBA [5]. Furthermore, a model is emerging where uncontrolled sympathetic and poor vagus control interacts with the microbiome and immune system to predispose individuals for psychiatric and gastrointestinal disease [7, 8].

Approximately 60% of those who seek medical care for FGIDs suffer from psychiatric illness [9]. Depression and anxiety disorders are the most common psychiatric co-morbidities in patients with FGIDs [9, 10]. Correspondingly, patients with high levels of depression or anxiety have a two-fold risk of developing IBS and patients with major depressive disorder (MDD) more frequently meet the criteria for IBS than healthy controls [11, 12]. Anxiety levels appear to be related to the burden of gastrointestinal symptoms and co-morbidity in IBS is observed in patients with panic disorder and generalised anxiety disorder [13–16]. High levels of anxiety might also be an independent predictor of developing FGIDs [17]. However, the same study also found higher levels of depression and anxiety at follow-up in patients with FGIDs at baseline, which is consistent with bidirectional brain-gut dysfunction.

Contradictory results have been found for subtypes of IBS in levels of depression compared with controls. One meta-analysis reported that patients with both diarrhoea-predominant IBS (IBS-D) and constipation-predominant IBS (IBS-C) showed higher levels of anxiety, whereas patients with only IBS-D appear to have higher levels of depression [18]. In contrast, another recent meta-analysis found that both depression and anxiety levels were higher in all subtypes of IBS as compared with controls, with the highest level of depression seen in patients with IBS-C [19].

IBS is characterised by unexplained chronic abdominal discomfort and pain associated with altered stool consistency and emptying [16, 20]. The prevalence of IBS varies widely in the general population because of regional and diagnostic differences, with a pooled global prevalence of 11.2%. Most studies show a higher prevalence in women than in men [21]. Because of the burden associated with abdominal and psychological symptoms, IBS can result in a profound reduction in health-related quality of life [3].

The complex aetiology of IBS remains elusive. Altered brain-gut interactions, increased gut permeability and immune activation may all be involved in the pathophysiology of IBS, at least in sub-populations of IBS patients [22, 23]. In addition, psychological, social and genetic factors may play a role in the development of IBS, with or without psychiatric comorbidity (e.g., through alterations of the HPA axis, increased visceral perception and psychological vulnerability) [10, 24]. The enigmatic influence of the bacterial flora on the brain and gut function remains an open question but may exert a major contribution to the brain-gut communication in IBS [25–27].

Pharmacological management of IBS seeks to ameliorate the predominant symptoms and reduce pain and discomfort. Illuminating the mechanisms of IBS with or without psychiatric co-morbidities is crucial to understand the pathophysiology and identification of new therapeutic targets in IBS [24]. Importantly, psychotherapy and antidepressants (such as selective serotonin reuptake inhibitors, SSRIs and tricyclic antidepressants, TCAs) have shown efficacy in different subpopulations of IBS [22, 28].

Although the link between anxiety, mood disorders and gastrointestinal symptoms has attracted attention, most studies have investigated the prevalence of psychiatric co-morbidity in patients with IBS. Regrettably, less research has been conducted from a psychiatric viewpoint, especially in young adult patients with a different disease panorama than older populations.

## Aim

The study aimed to investigate the relationship between gastrointestinal symptoms, depressive symptoms and trait anxiety in a cohort of young adult patients seeking psychiatric care and to compare these patients with healthy controls. The main hypothesis was that there might be a positive association between the severity of subjective depressive and gastrointestinal symptoms in young adults seeking psychiatric care. Specifically, we aimed to i) compare the severity of gastrointestinal symptoms between psychiatric patients and healthy controls and between patients with and without current psychotropic medication, ii) investigate whether gastrointestinal symptom burden is related to depressive symptom severity and iii) explore the role of trait anxiety in this context.

## Methods

The study was approved by the Regional Ethics Committee in Uppsala. All patients signed an informed consent form on recruitment to the study. The authors assert that all procedures contributing to this work comply with the ethical standards of the relevant national and institutional committees on human experimentation and with the Helsinki Declaration of 1975, as revised in 2008.

### Sample size

A power calculation was conducted using G*power version 3.1, to determine the required sample size. The difference in proportions was set to 0.19 (i.e. p2 - p1 = 0.30–0.11) as the prevalence of IBS in patients with depression has previously been estimated to be 30% [29] and in the general population around 11% [21]. With α = 0.05 and a desired power of 80%, the minimum required sample size in each group was calculated to be 70.

### Participants

Uppsala Psychiatric Patient Samples (UPP) is a project for the collection of biological material from patients seeking psychiatric care at the Department of General Psychiatry at Uppsala University Hospital, Sweden [30]. This study included patients with any psychiatric disorders, aged 18–25, recruited to UPP between January 2013 and December 2017 after the introduction of the Gastrointestinal Symptom Rating Scale for IBS (GSRS-IBS) to the protocol (*n* = 682). Assessment of psychiatric diagnoses was performed by trained medical doctors or clinical psychologists using the Structured Clinical Interview for DSM IV axis I disorders (SCID-I) [31] or the Swedish version of the Mini-International Neuropsychiatric Interview (M.I.N.I) [32]. Both instruments are standard in clinical practice and they display good overlap [32]. Exclusion criteria i) Patients who did not undergo structured psychiatric assessment were excluded (*n* = 27), ii) patients for which symptoms assessment questionnaires were incomplete (*n* = 91) iii), more than 6 months had passed between questionnaires and diagnostic interviews (*n* = 36) and iv) established co-morbidity with inflammatory bowel disorder (*n* = 12), endometriosis (*n* = 3) and anorexia nervosa (*n* = 22). The final sample was comprised of 491 patients.

Controls (*n* = 139) under the age of 30 years included staff from Uppsala University Hospital and students at Uppsala University. Controls who met the criteria for any current axis I diagnosis (*n* = 14), diagnosed with endometriosis (*n* = 1) or who scored high on questionnaires indicating alcohol or substance abuse (*n* = 8) were excluded. Cases with incomplete or missing questionnaires were also excluded from statistical analysis (*n* = 31). The final sample included 85 healthy controls.

### Questionnaires

The Swedish translation of the GSRS-IBS, a validated self-assessment instrument for the evaluation of IBS symptoms was used to measure gastrointestinal symptoms [33]. The patients completed the GSRS-IBS at one time point, reporting gastrointestinal symptoms from the past week on a seven-point [1–7] Likert-scale, ranging from 1 = “no discomfort at all” to 7 = “very severe discomfort”. The total possible score ranged from a low of 13 to a high of 91 points. The instrument’s questions are grouped into five symptom clusters: pain (question 1 and 2), bloating (question 3, 4 and 13), constipation (question 5 and 8), diarrhoea (question 6, 7, 9 and 10) and satiety (question 11 and 12). The GSRS-IBS total score have high discriminant and convergent validity and can be used to measure IBS symptom severity also in the general population [34],

The self-rating version of the Montgomery-Åsberg Depression Rating Scale (MADRS-S) was used to rate depressive symptoms [35–37]. The MADRS-S has been demonstrated to be a reliable patient-administered tool for depressive symptoms. The instrument consists of nine questions rated on a six-point (0–6) Likert-scale, with a possible total score ranging from 0 to 54 [38].

The Swedish universities Scales of Personality (SSP) is a revised and shortened version of the Karolinska Scales of Personality with improved psychometric properties [39]. This self-assessment tool consists of 91 items divided into 13 scales to assess aspects of personality traits. Three of these scales were used to assess trait anxiety: somatic trait anxiety (STA), psychic trait anxiety (PsTA) and stress susceptibility (SS). An example of an item from PsTA is: “I’m the kind of person who is excessively sensitive and easily hurt”. Each item is rated on a four-point scale ranging from “Does not apply at all” to “Applies completely”. Previous studies report good psychometric properties for the SSP, with satisfactory internal consistency, agreement with personality constructs of other instruments and similar factor loadings between different samples [39, 40]. In a Swedish normative sample, the Cronbach’s alpha coefficients ranged from 0.74 to 0.82 for the STA, PsTA and SS scales [39]. In an Estonian sample, the STA, PsTA and SS scales were moderately to strongly correlated with the neuroticism factor of the Revised NEO Personality Inventory scales [40]. A computerised script was used to transform SSP mean scores to normative T-scores in accordance with the instructions of the SSP manual (version 2.1).

Finally, study participants also filled out questionnaires on sociodemographic and medical history.

### Data analysis

All statistical analyses were conducted with the Statistical Package for the Social Sciences (SPSS) Statistics Version 25.0 and R Studio (version 1.2.1335) for visualisations. Before data analysis, the normal distribution of the continuous variables (total GSRS-IBS, MADRS-S and SSP scale scores) was assessed using the histogram, Q-Q plots and box-plots and tested with the Shapiro-Wilk test of normality (*p* > 0.05). For the GSRS-IBS scores, non-parametric tests were used because of non-normally distributed data. Differences between groups were analysed using the Mann-Whitney U test and for categorical comparisons Chi square tests or Fisher’s exact tests were used. Gastrointestinal symptom severity was compared between patients and healthy controls with a Mann-Whitney U test. To assess whether the effect was dependent on medication gastrointestinal symptom severity was then compared between three groups (patients with ongoing psychotropic medication, psychotropic-medication-free patients, healthy controls) using the Kruskal-Wallis one-way analysis of variance. Correlations between the severity of depressive symptoms (MADRS-S) and GSRS-IBS scores were investigated with Spearman’s rank coefficients in patients and controls (separately and combined). To control for possible confounders a generalized linear model was constructed using Gamma log link distribution, with the total GSRS-IBS score as the dependent variable and the total MADRS-S scores and BMI as independent variables. Bulimia nervosa was suspected to impact both variables of interest (MADRS-S scores and GSRS-IBS scores), which was confirmed in a bivariate correlation analysis, and was therefore added to the model as an independent variable.

The impact of trait anxiety (STA, PsTA and SS) on gastrointestinal symptom burden was assessed using Spearman’s rank coefficients. Significant correlations emerged between all three scales and the GSRS-IBS total score. These scales were then added to the generalized linear model to examine the robustness of the correlation between the total GSRS-IBS score and the total MADRS-S score. The individual depressive symptoms (MADRS-S items) and IBS symptom clusters (abdominal pain, diarrhoea, bloating, early satiety and constipation) were further explored using Spearman’s rank coefficients. Significance was defined as *p* < 0.05.

To determine potential subgroups in the patient population a principal component analysis (PCA) and cluster analysis were conducted. First, normalised z-scores of the individual items of the MADRS-S and GSRS-IBS scales and the T-scores for the scales of STA, PsTA, SS as well as BMI were used to identify principal components, which accounted for most of the variance in the dataset. A two-step cluster analysis was performed in which the number of clusters to be generated was not specified in advance.

## Results

### Participant characteristics

Participant characteristics are summarised in Table 1. Half of the patients met the criteria for a current depressive episode (52.3%) and nearly two thirds (64.6%) suffered from any anxiety disorder as defined by the DSM-IV (generalised anxiety disorder, social anxiety disorder, agoraphobia, post-traumatic stress disorder or panic disorder). Most (89.4%) of the patients and 9.4% of the controls had a lifetime history of a depressive episode. Co-morbidity was high in the patient group and many had more than one psychiatric diagnosis.

**Table 1:** Study participant characteristics (*N* = 576)

	Patients (***n*** = 491)	Controls (***n*** = 85)	***P***-value
**Women, n (%)**	361 (73.5)	62 (72.9)	ns
**Age, mean (SD)**	21.5 (2.2)	25.2 (2.3)	**
**BMI, mean (SD)**	23.8 (5.4)	22.7 (3.2)	ns
**MADRS-S, median (range)**	23 (0–49)	4 (0–20)	**
**GSRS-IBS, median (range)**	30 (13–79)	22 (13–70)	**
**Medication, n (%)**			
Antidepressant, any	263 (53.6)	3 (3.5)^a^	**
Psychotropic medication, any	342 (69.7)	4 (4.7)^b^	**
SSRI	192 (39.1)	2 (2.4)	**
**Diagnosis, n (%)**			
Current depressive episode (UP/BP)	257 (52.3)	0	**
Recurrent MDD	184 (37.5)	0	**
Lifetime history of depressive episodes (UP/BP)	439 (89.4)	8 (9.4)	**
Bipolar disorder, any	91 (56.0)	0	**
Anxiety disorder, any	317 (61.2)	0	**

**p < 0.01 level, ns = Non-significant, ^a^ One control with ongoing SNRI treatment and two controls with SSRI. ^b^ One control with anti-epileptic medication. Abbreviations: *SD* standard deviation, *MADRS-S* Montgomery-Åsberg Depression Rating Scale Self-assessment, *GSRS-IBS* Gastrointestinal Symptoms Rating Scale for Irritable Bowel Syndrome, *SSRI* selective serotonin re-uptake inhibitor, *MDD* Major Mood Disorder, *UP/BP* Unipolar/Bipolar

### Gastrointestinal symptoms, depressive symptoms and trait anxiety

**Fig. 1:**
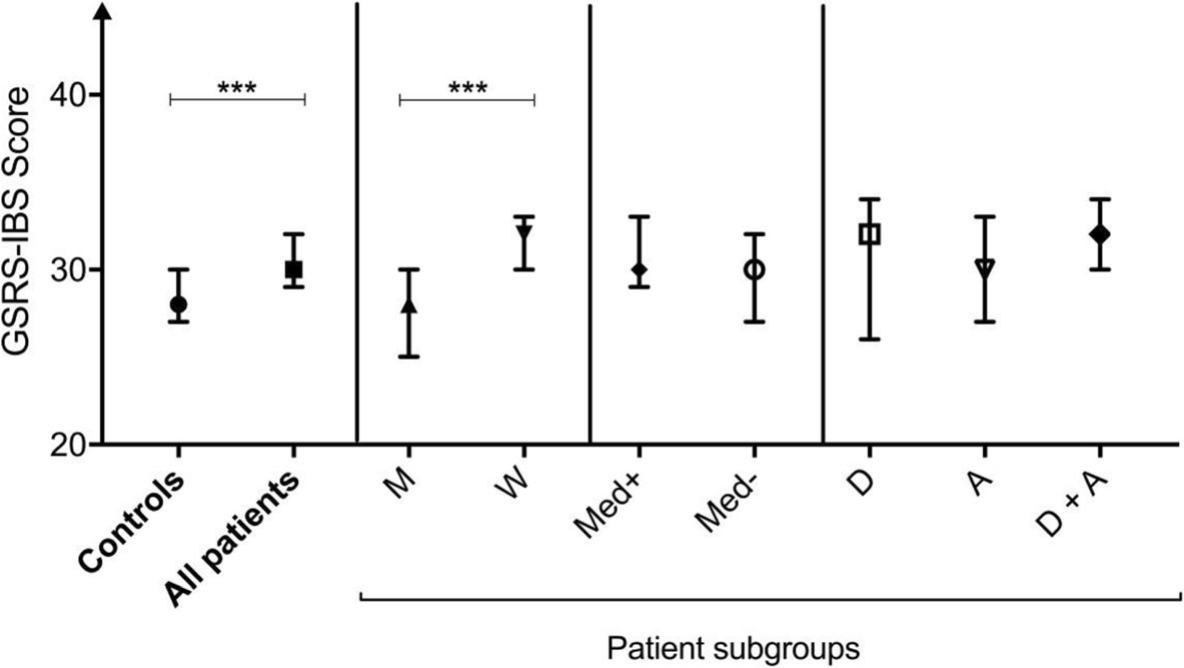
Gastrointestinal symptoms in controls and patients: Symptoms are measured by total Gastrointestinal Symptoms Rating Scale for Irritable Bowel Syndrome (GSRS-IBS) score. Patients are grouped according to gender, medication state and diagnosis (current depressive episode, any anxiety disorder, or both). Abbreviations: M = men, W = women, Med + = patients with psychotropic medication, Med- = patients without psychotropic medication, D = Current depressive episode, A = Any anxiety disorder, D + A = Current depressive episode and Any anxiety disorder; *** *p* < 0.001

Patients reported more gastrointestinal symptoms, defined as higher total scores on the GSRS-IBS, than controls (median 30, range 13–79 vs. median 22 range 13–70, *p* < 0.001). In a subgroup analysis, controls had lower total GSRS-IBS scores than patients with psychotropic medication (*n* = 342, median 31, range 13–78, *p* < 0.001) and without psychotropic medication (*n* = 149, median 30, range 13–79, *p* < 0.001). The difference in GSRS-IBS total score was significant in both women and men, with women in the patient group reporting significantly higher total GSRS-IBS scores than men (median 32, range 13–79 vs. median 28, range 13–72, *p* < 0.001), see Fig. 1.

The total GSRS-IBS score was positively correlated with the total MADRS-S-score in patients (*ρ* = 0.290, *p* < 0.001) and controls (*ρ* = 0.482, *p* < 0.001). In an exploratory analysis all the items of the MADRS-S had significant weak to moderate correlations with all the individual symptom clusters of the GSRS-IBS (Table 2).

**Table 2:** Exploratory analysis of correlations between symptoms: MADRS-S items correlate with IBS symptom clusters (all participants and controls are included, *N* = 576). Significant Spearman’s Rho correlation coefficients > 0.3 are marked in bold

	Pain	Bloating	Constipation	Diarrhoea	Satiety	Total GSRS-IBS score
**1. Mood**	0.183***	0.167***	0.194***	0.187***	0.248***	0.255***
**2. Feelings of unease**	0.316***	0.270***	0.239***	0.282***	0.271***	0.362***
**3. Sleep**	0.210***	0.208***	0.194***	0.238***	0.259***	0.292***
**4. Appetite**	0.170***	0.194***	0.132**	0.184***	0.407***	0.287***
**5. Ability to concentrate**	0.223***	0.251***	0.253***	0.250***	0.284***	0.331***
**6. Initiative**	0.262***	0.235***	0.218***	0.267***	0.306***	0.338***
**7. Emotional involvement**	0.202***	0.213***	0.165***	0.202***	0.301***	0.285***
**8. Pessimism**	0.221***	0.241***	0.214***	0.253***	0.263***	0.317***
**9. Zest for life**	0.185***	0.148***	0.118**	0.221***	0.236***	0.252***
**MADRS-S total score**	0.287***	0.279***	0.246***	0.295***	0.368***	0.391***

** *p* < 0.01, *** *p* < 0.001; Abbreviations: *GSRS-IBS* Gastrointestinal Symptoms Rating scale for Irritable Bowel Syndrome, *MADRS-S* Montgomery-Åsberg Depression Rating Scale - Self assessment

The relationship between total GSRS-IBS scores and MADRS-S was further analysed in a generalized linear model and remained significant after adjusting for sex, BMI and ongoing bulimia nervosa (Table 3).

**Table 3:** Generalized linear models for the total GSRS-IBS scores and MADRS-S total score in young adult patients (*n* = 491)

	B	SE	P
**Model 1**			
** MADRS-S total score**	0.013	0.0018	**< 0.001**
**Sex (women)**	0.143	0.0385	**< 0.001**
**BMI**	−0.007	0.0031	**0.027**
**Bulimia nervosa**	0.111	0.0086	0.075
**Model 2**			
** MADRS-S total score**	0.007	0.0020	**< 0.001**
** Sex (women)**	0.134	0.0371	**< 0.001**
** BMI**	−0.007	0.0030	**0.024**
**Bulimia nervosa**	0.144	0.0588	**0.014**
**STA**	0.012	0.0019	**< 0.001**
**PsTA**	−0.007	0.0021	**0.002**
**SS**	0.005	0.0018	**0.002**

Abbreviations: *GSRS-IBS* Gastrointestinal Symptoms Rating Scale for Irritable Bowel Syndrome, *SE* standard error, *MADRS-S* Montgomery-Åsberg Depression Rating Scale - Self assessment, *BMI* body mass index, *STA* Somatic trait anxiety, *PsTA* Psychic trait anxiety, *SS* Stress susceptibility

The influence of trait anxiety on GSRS-IBS scores was first analysed in a bivariate correlation analysis in which significant weak to moderate positive correlations were found between the total GSRS-IBS score and the scores of the three SSP scales: STA (*ρ* = 0.313, *p* < 0.001), PsTA (*ρ* = 0.147, *p* = 0.001) and SST (*ρ* = 0.233, p < 0.001). A second generalized linear model was constructed in which STA, PsTA and SS were included as independent variables. Total MADRS-S score and all factors included in the model proved to be independent predictors of the total GSRS-IBS score (Table 3). In addition, all of the three SSP scale scores were significantly correlated with all IBS symptom clusters. The correlations between the STA scale score and the IBS symptom clusters were stronger than for the PsTA and the SS scale score and there was a marginally larger effect size for the symptoms of abdominal pain and early satiety (Supplementary Table 1).

### Patient subgroups identified by PCA and cluster analysis

**Fig. 2:**
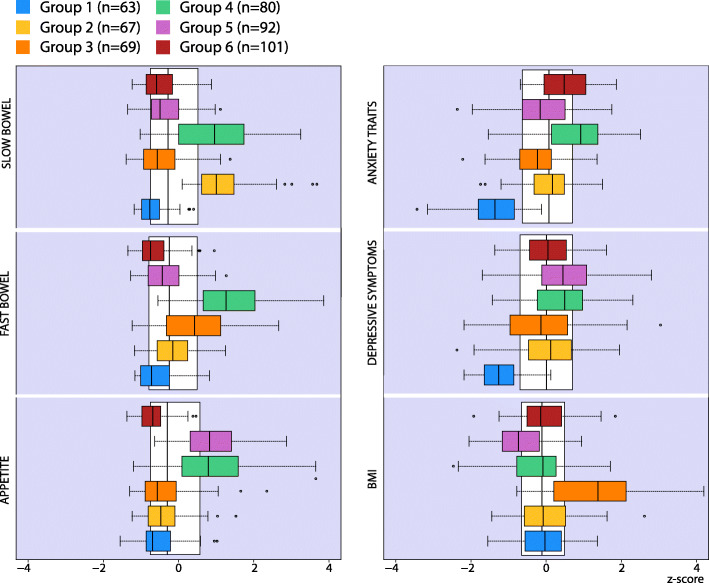
Boxplot showing the six cluster groups: The groups are shown in relation to the six factors extracted from the principal component analysis

Using PCA, six factors were extracted (eigenvalues > 1), explaining 63.9% of the total variance. Only variables loading higher than 0.4 were included in the PCA, see supplementary Table 2. The Kaiser-Meyer-Olkin measure of sampling adequacy was 0.880, demonstrating sampling accuracy, and Bartlett’s test of sphericity was significant (*p* < 0.001). The six factors were characterised according to the items that loaded the highest on each factor. The factors were labelled as follows: slow bowel, fast bowel, depressive symptoms, trait anxiety, disturbed appetite and BMI. In the cluster analysis six cluster groups were identified, ranging from 63 to 101 individuals in each cluster. The cluster outcome showed a silhouette measure of cohesion and separation of 0.30, indicating fair cluster quality. Characteristics of these six clusters are depicted in Fig. 2. Patients in cluster 1 (*n* = 63) did not score high on any of the factors. Cluster 2 patients (*n* = 67) were largely characterised by high scores for slow bowel. The patients in cluster 3 (*n* = 69) were distinguished by high scores for fast bowel and a high BMI. Cluster 4 patients (*n* = 80) reported numerous symptoms (fast and slow bowel, disturbed appetite with high anxiety traits and depressive symptoms). Cluster 5 patients (*n* = 93) were mainly marked by disturbed appetite and low BMI as well as an overall high depressive symptoms score. Cluster 6 (*n* = 101) included patients with mainly high anxiety traits but low scores regarding fast or slow bowel and disturbed appetite.

## Discussion

This study examined the prevalence of gastrointestinal symptoms and their relationship to depressive symptoms and anxiety traits in young adults seeking outpatient psychiatric care. We found a larger burden of gastrointestinal symptoms in patients compared with healthy controls. Gastrointestinal symptom severity was positively associated with overall depressive symptom burden and the severity of individual depressive symptoms. Consistent with previous results [41], trait anxiety correlated with gastrointestinal symptom burden independent of the effects from depressive symptom severity, bulimia nervosa gender and BMI.

Patients with IBS are known to have a high psychiatric co-morbidity. This connection between IBS and psychiatric co-morbidity appears to be bidirectional, with a high prevalence of IBS diagnosis and IBS symptoms in psychiatric patients, especially in patients with mood and anxiety disorders [11, 29]. In this study, gastrointestinal symptom burden correlated with depressive symptom severity, a finding in accordance with previous evidence [42]. One large Korean study, however, reported that IBS was more common in patients with mild depression than in those with severe depression [43]. Our current study included only outpatients and therefore may not generalise to inpatients with severe depression. The correlation between the total MADRS-S score and the total GSRS-IBS score in our study showed a larger effect size in controls than in patients. Exaggerated gastrointestinal symptoms may cause a greater impact on mood and function in an otherwise healthy individual than in a psychiatric patient who also suffers from depression or anxiety.

We identified different subgroups within the studied patient population. One subgroup of patients (cluster 4) had a high level of gastrointestinal symptoms (fast and slow bowel and disturbed appetite) together with high trait anxiety and high levels of depressive symptoms. In contrast to cluster 4, patients in cluster 1 did not score high on any of the factors and thus they may constitute a different subgroup of patients, perhaps closer to remission, as it has been shown that patients in remission from recurrent depression do not have more symptoms of IBS than healthy controls [42].

In our study, personality traits reflecting anxiety and stress susceptibility significantly correlated with gastrointestinal symptom burden with the largest effect size for somatic trait anxiety, which is consistent with the proposed presence of somatisation in patients with IBS [44]. In animal models, central pathways mediating stress and anxiety have been linked to increased gut sensitivity [45]. In patients with IBS, visceral hypersensitivity is a result of several factors, including increased afferent signalling to the brain [46], abnormal descending modulation of pain, and dysfunction of the medial pain system, which is responsible for the emotional aspect of pain [47]. Immune activity in the intestinal mucosa or the central nervous system may play a role in the development of visceral hypersensitivity. However, the precise role of immune activity remains contentious [48, 49].

The pivotal question of the causation of disease is the temporal relationship of the onset of symptoms or the making of a classified diagnosis. The use of questionnaires does not focus on this relationship and the long-term observation time needed precludes conclusions as regards to the onset of disease or symptoms. Animal models may be useful to reveal possible etiological factors related to the onset of gastrointestinal symptoms [50–52].

Different theories have been proposed to explain the high co-morbidity of IBS and psychiatric illness in which the GBA plays a central role in mediating this interaction. Alterations of the autonomic nervous system with increased sympathetic and decreased parasympathetic activity can influence perception from the gastrointestinal tract [53]. Local hormones and inflammation further influence the autonomic nervous system and the vagus nerve may provide a transport route for microbial and other metabolites to the CNS [7, 8]. Dysregulation of the HPA axis has also been observed in female IBS patients of which both basal levels of cortisol and stress-induced cortisol levels were elevated compared with healthy controls [54]. Moreover, alterations in the gut microbiome have been central in seeking to understand the complex relationship between depression and IBS [55]. Significant differences in microbiota composition have been demonstrated in patients with IBS compared with controls [56]. The intestinal microbiota can produce neurologically active substances (e.g., gamma-aminobutyric acid and short-chain fatty acids, SCFAs) that can influence immune regulation and the mucosal barrier, but their role in stress-induced behavioural and physiological alterations is poorly understood [57, 58]. In mice, administration of SCFAs was found to alleviate stress-induced anhedonia and increased responsiveness to acute stress as well as reversed changes in intestinal permeability caused by psychosocial stress [59]. Moreover, breakdown of the mucosal barrier in the gut wall, bacterial translocation and immune activation are believed to result in excessive cytokine production that can affect not only gut functions and the intestinal microflora, but also brain functions and behaviour [60, 61]. Elevated pro-inflammatory markers, especially interleukin-1, interleukin-6 and tumour necrosis factor-alpha are seen in patients with depression and we already know that inflammation may induce depression [62].

A limitation of this cross-sectional study is that the patient group is not delimited to a single diagnosis; rather, symptomatic measurements of depression and anxiety levels are analysed, which restricts the possibility to examine differences between individual diagnostic groups. Still, because of the overlap in concomitant diagnoses, specific psychiatric symptoms reflecting different underlying biological mechanisms are probably more relevant. For this reason, the MADRS-S total score, as a measure of the level of depressive symptoms, and the scales of the SSP were used rather than the diagnostic group affiliation to capture these symptoms or behaviours. Here, we wished to investigate the impact of trait anxiety, which is why individual diagnoses of anxiety were not further analysed in the context of gastrointestinal symptoms. Another limitation is that patients completed the SSP questionnaires with ongoing depressive symptoms and a possible state effect cannot be ruled out. Additionally, the control group was significantly older than the patient group, which may have influenced the results.

Gastrointestinal symptoms in this study are self-reported. The GSRS-IBS questionnaire was developed to evaluate treatment for IBS and in our study the total score is used as a measurement of symptom burden. This method is likely more inclusive, whereas when the actual diagnosis is used rather than reported symptoms, the propensity to seek medical care must be considered. While the GSRS-IBS total score reflects symptom burden, gastrointestinal symptoms may vary during each week (such as alternating diarrhoea and constipation), which influences the comparison of the total score between individuals. An attempt to visualise this issue is through the cluster analysis in which different groups with a diverse spectrum of symptoms were identified.

Studies have found ameliorated IBS symptoms with SSRI treatment, but also with tricyclic antidepressants [63, 64]. Of note, there were no differences in gastrointestinal symptom burden between patients with and without psychotropic medication (antidepressants, including SSRI’s). Indeed, treatment with SSRIs for IBS may not be a “one size fits all” solution, but rather a treatment that only a subpopulation of IBS patients benefits from. In fact, one study reported more IBS symptoms in patients with SSRI treatment [42]. The present study design did not allow for distinction from GI side effects from SSRI treatment, why possible benefits may have been cancelled out. Also, medication compliance could not be fully determined in this study, which could have influenced the results.

The prevalence of FGIDs is high in patients with eating disorders [65]. Patients who met the criteria for anorexia nervosa were excluded because this diagnosis constitutes a very special catabolic state due to starvation, making results difficult to interpret and generalise. Patients with ongoing bulimia were included, although they generally have a high level of self-starvation, but this potential confounder was adjusted for in the generalized linear model and did not influence the results.

Young adults seeking psychiatric care reported more gastrointestinal symptoms than controls, regardless of ongoing psychotropic medication. Gastrointestinal symptoms correlated positively with the severity of depressive symptoms and trait anxiety. Although we confirmed significant associations between gastrointestinal and psychiatric symptoms their aetiology is multifactorial, complex and not well understood. The cluster analysis revealed different groups with a diverse spectrum of symptoms. We believe this may be important for designing future studies focused on understanding biological factors where genetic vulnerability and disease mechanisms may differ between clusters. The high co-morbidity of mood and anxiety disorders and gastrointestinal symptoms and IBS in early adult life motivates further investigation to identify common denominators in the complex mechanisms underlying these disorders.

## Supplementary Information

**Additional file 1: Supplementary Table 1.** Bivariate correlations between GSRS-IBS symptom clusters and SSP scales. Correlation coefficients >0.3 are marked in bold (*N*=576). **Supplementary Table 2.** Principle component loading scores of each individual Montgomery-Åsberg Depression Rating Scale- Self assessment MADRS-S, Gastrointestinal Symptom Rating Scale for Irritable Bowel Syndrome (GSRS-IBS), and trait anxiety scales; Somatic trait anxiety (STA) Psychic trait anxiety (PsTA) and Stress susceptibility (SS) as well as BMI (z-scores). Six factors were extracted with eigenvalues > 1, explaining 63.9% of the total variance. Only variables loading higher than 0.4 were included. According to the items that loaded the highest on each factor they were labelled as follows: slow bowel, fast bowel, depressive symptoms, trait anxiety, disturbed appetite and BMI (*n* = 491).

## Data Availability

The data that support the findings of this study are available from the corresponding author upon request in accordance with the General Data Protection Regulation.

## Abbreviations

BMI: Body Mass Index
FGIDs: Functional gastrointestinal disorders
GBA: Gut-brain axis
GI: Gastrointestinal
GSRS-IBS: Gastrointestinal Symptom Rating Scale for Irritable Bowel Syndrome
HPA: Hypothalamic-pituitary-adrenal
IBS: Irritable bowel syndrome
IBS-C: Constipation-predominant IBS
IBS-D: Diarrhoea-predominant IBS
MADRS-S: Montgomery-Åsberg Depression Rating Scale- Self assessment
MDD: Major depressive disorder
PCA: Principal component analysis
PsTA: Psychic trait anxiety
SCFAs: Short-chain fatty acids
SCID-I: Structured Clinical Interview for DSM IV axis I disorders
SPSS: Statistical Package for the Social Sciences
SS: Stress susceptibility
SSP: Swedish universities scales of Personality
SSRIs: Selective serotonin reuptake inhibitors
STA: Somatic trait anxiety
TCAs: Tricyclic antidepressants
UPP: Uppsala Psychiatric Patient Samples
