# Supplementary material for: A cross-sectional study of gastrointestinal symptoms, depressive symptoms and trait anxiety in young adults
Source: BMC Psychiatry. 2020 Nov 11;20:535. doi: 10.1186/s12888-020-02940-2 (PMC7661167; doi:10.1186/s12888-020-02940-2)
Supplement: Supplementary file 1 — Additional file 1: Supplementary Table 1. Bivariate correlations between GSRS-IBS symptom clusters and SSP scales. Correlation coefficients >0.3 are marked in bold (N=576). Supplementary Table 2. Principle component loading scores of each individual Montgomery-Åsberg Depression Rating Scale- Self assessment MADRS-S, Gastrointestinal Symptom Rating Scale for Irritable Bowel Syndrome (GSRS-IBS), and trait anxiety scales; Somatic trait anxiety (STA) Psychic trait anxiety (PsTA) and Stress susceptibility (SS) as well as BMI (z-scores). Six factors were extracted with eigenvalues > 1, explaining 63.9% of the total variance. Only variables loading higher than 0.4 were included. According to the items that loaded the highest on each factor they were labelled as follows: slow bowel, fast bowel, depressive symptoms, trait anxiety, disturbed appetite and BMI (n = 491). [file 12888_2020_2940_MOESM1_ESM.docx]

**Supplementary Table 1.** **Bivariate correlations between GSRS-IBS symptom clusters and SSP scales.** Correlation coefficients >0.3 are marked in bold (N=576).

|  | **STA** | **PsTA** | **SS** |
| --- | --- | --- | --- |
| Satiety | **0.349***** | 0.210*** | 0.251*** |
| Diarrhoea | **0.318***** | 0.184*** | 0.268*** |
| Constipation | 0.223*** | 0.114** | 0.249*** |
| Bloating | **0.309***** | 0.160*** | 0.256*** |
| Pain | **0.350***** | 0.184*** | 0.288*** |
| Total GSRS-IBS score | **0.401***** | 0.227*** | **0.342***** |

*** p<0.001;

Abbreviations: GSRS-IBS = Gastrointestinal Symptoms Rating scale for Irritable Bowel Syndrome, SSP = Swedish universities Scales of Personality, STA = Somatic trait anxiety, PsTA = Psychic trait anxiety, SS = Stress susceptibility, GSRS-IBS = Gastrointestinal Symptoms Rating scale for Irritable Bowel Syndrome.

**Supplementary Table 2:** Principle component loading scores of each individual Montgomery-Åsberg Depression Rating Scale- Self assessment MADRS-S, Gastrointestinal Symptom Rating Scale for Irritable Bowel Syndrome (GSRS-IBS), and trait anxiety scales; Somatic trait anxiety (STA) Psychic trait anxiety (PsTA) and Stress susceptibility (SS) as well as BMI (z-scores). Six factors were extracted with eigenvalues >1, explaining 63.9 % of the total variance. Only variables loading higher than 0.4 were included. According to the items that loaded the highest on each factor they were labelled as follows: slow bowel, fast bowel, depressive symptoms, trait anxiety, disturbed appetite and BMI (n=491).

| **Pattern Matrix** | | | | | | |
| --- | --- | --- | --- | --- | --- | --- |
|  | **Component** | | | | | |
|  | **Slow bowel** | **Depressive symptoms** | **Fast bowel** | **Disturbed appetite** | **Trait anxiety** | **BMI** |
| **GSRS-Constipation** | ,907 |  |  |  |  |  |
| **GSRS-Hard stools** | ,883 |  |  |  |  |  |
| **GSRS-Not emptying** | ,808 |  |  |  |  |  |
| **GSRS-Distension** | ,574 |  |  |  |  |  |
| **GSRS-Bloating** | ,566 |  |  |  |  |  |
| **GSRS-Abdominal Pain** | ,458 |  |  |  |  |  |
| **MADRS-S-Zest for Life** |  | ,854 |  |  |  |  |
| **MADRS-S-Mood** |  | ,853 |  |  |  |  |
| **MADRS-S-Emotional involvement** |  | ,770 |  |  |  |  |
| **MADRS-S-Pessimism** |  | ,690 |  |  |  |  |
| **MADRS-S-Initiative** |  | ,687 |  |  |  |  |
| **MADRS-S-Feeling of unease** |  | ,508 |  |  |  |  |
| **MADRS-S-Appetite** |  | ,501 |  | ,443 |  |  |
| **MADRS-S-Ability to concentrate** |  | ,456 |  |  |  |  |
| **MADRS-S-Sleep** |  |  |  |  |  |  |
| **GSRS-Loose stools** |  |  | ,875 |  |  |  |
| **GSRS-Diarrhoea** |  |  | ,861 |  |  |  |
| **GSRS-Urgency** |  |  | ,761 |  |  |  |
| **GSRS-Relief by defacation** |  |  | ,572 |  |  |  |
| **GSRS-Passing gas** |  |  | ,416 |  |  |  |
| **GSRS-Feeling full long after food** |  |  |  | ,885 |  |  |
| **GSRS-Early satiety** |  |  |  | ,859 |  |  |
| **PsTA** |  |  |  |  | -,812 |  |
| **SS** |  |  |  |  | -,804 |  |
| **STA** |  |  |  |  | -,670 |  |
| **BMI** |  |  |  |  |  | ,908 |
